# Effectiveness of Yinhua Pinggan granules in community-acquired pneumonia: a randomized, double-blind clinical trial

**DOI:** 10.3389/fphar.2025.1446319

**Published:** 2025-03-04

**Authors:** Jiao-Li Wang, Hao-Ran Hu, Yi-Lei Guo, Jin Han, Hai-Tong Wan, Yu-Xiao Tong, Man Luo, Xian-Wen Li

**Affiliations:** ^1^ College of Basic Medical Science, Zhejiang Chinese Medical University, Hangzhou, China; ^2^ Department of Respiratory Medicine, Affiliated Hangzhou First People’s Hospital, School of Medicine, Westlake University, Hangzhou, China; ^3^ Academy of Chinese Medical Sciences, Henan University of Chinese Medicine, Zhengzhou, China; ^4^ School of Nursing, Hangzhou Medical College, Hangzhou, China

**Keywords:** community-acquired pneumonia, Yinhua Pinggan granules, traditional Chinese medicine, cure rate, randomized controlled trial

## Abstract

**Ethnopharmacological relevance:**

Community-acquired pneumonia (CAP) is an acute inflammation of the alveoli and distal bronchi caused by bacterial, viral, or other pathogenic microbial infections. Yinhua Pinggan (YHPG) granules have demonstrated anti-inflammatory, antibacterial, and antiviral effects, suggesting their potential as a treatment option for CAP.

**Aim:**

To assess the efficacy and safety of traditional Chinese medicine (TCM), YHPG granules, in combination with conventional pneumonia treatments.

**Materials and methods:**

This randomized, double-blind, placebo-controlled clinical trial was conducted at a medical center in Hangzhou and involved 240 eligible participants. In addition to conventional pneumonia treatment, participants were randomly assigned in a 1:1 ratio to receive either YHPG granules or placebo for 10 days. The primary outcome measure was the difference in pneumonia cure rates at the end of treatment. Secondary outcomes included chest CT absorption rate, criticality score (SMART-COP score), Acute Physiology and Chronic Health Evaluation II (APACHE II) score, C-reactive protein (CRP) level, lactate (LC) level, procalcitonin (PCT) level, time for symptom recovery, length of hospital stay, and TCM syndrome scores.

**Results:**

In total, 229 participants were included in the analysis. The pneumonia cure rate in the YHPG granule group was higher than that in the placebo group (37.2% vs. 22.4%, mean difference: 14.75%, 95% CI: 3.05–26.46, *p* < 0.05), indicating the superiority of YHPG granules. The granules significantly improved the chest CT absorption rate, pneumonia severity, and CRP and LC levels (*p* < 0.05). Additionally, YHPG granules resulted in a shorter recovery time from fever and lung rales, reduced hospital stay, and lowered the TCM syndrome scores than the placebo (*p* < 0.05). No significant differences were observed in other outcomes between the two groups (*p* > 0.05). Notably, the use of YHPG granules was associated with fewer adverse reactions.

**Conclusion:**

YHPG granules are a promising adjunct therapeutic agent for CAP.

**Clinical Trial Registration:**

https://www.chictr.org.cn/showproj.html?proj=127908, identifier ChiCTR2100047501.

## 1 Introduction

Pneumonia is acute inflammation of the alveoli and distal bronchi caused by bacterial, viral, or other pathogenic microbial infections (Torres et al., 2021). It is generally categorized as community-acquired (CAP) or hospital-acquired pneumonia (HAP; Torres et al., 2021). According to the 2019 Global Burden of Disease Study, lower respiratory tract infections, including pneumonia, are a common cause of global mortality (GBD, 2019 Diseases and Injuries Collaborators, 2020). Despite the varying mortality rates of CAP due to factors such as country, age, and sex, the overall fatality rate is between 2.5% and 20% (Heo and Song, 2018). Therefore, treatment of CAP is crucial. *Streptococcus pneumoniae* is the most common pathogen that causes CAP, followed by atypical pathogens and viruses (Cillóniz et al., 2011). Given the often unknown causative pathogens in the early stages of CAP, timely administration of empirical antibiotics remains pivotal in treatment. Although the condition of patients with CAP improves with the use of antibiotics, irrational antibiotic prescriptions pose risks such as heightened bacterial resistance (Costelloe et al., 2010) and adverse drug reactions (Lin et al., 2009). Therefore, it is necessary to identify adjunct therapeutic agents for CAP treatment.

Traditional Chinese botanical preparations are characterized by multiple metabolites, pathways, and targets, presenting a potential expansion of treatment options for CAP. Hence, the question of whether botanical drugs can effectively complement conventional treatments is of considerable interest. Yinhua Pinggan (YHPG) granules, originating from Ma Huang Tang (prescription from *Shang-Han-Lun*) or San Ao Tang (prescription from *Tai Ping Hui Min He Ji Ju Fang*), are notable for their effects on clearing heat, detoxifying, stopping cough, resolving phlegm, and relieving asthma. Recent research has highlighted the anti-inflammatory activity of YHPG granules, with rutin, quercetin, and ellagic acid identified as the three major active metabolites that effectively inhibit central pro-inflammatory genes (Jin et al., 2022). *In vitro* experiments have demonstrated the inhibitory effects of YHPG granules on common pathogenic bacteria in the respiratory tract, including *S. pneumoniae*, *Staphylococcus aureus*, *Klebsiella pneumoniae*, *Haemophilus influenzae*, and *Escherichia coli* (Wan et al., 2016). This study demonstrated the broad-spectrum antibacterial capabilities of YHPG granules, positioning them as a potential treatment for CAP primarily caused by bacterial infections. Previous studies have shown that YHPG granules can suppress lung tissue inflammation and viral load in mice infected with H1N1 influenza A virus, concurrently regulating immune function imbalances (Peng et al., 2015; Peng et al., 2016). Furthermore, evidence from a randomized controlled trial (RCT) suggests that YHPG granules can alleviate clinical symptoms in patients with respiratory viral infections, demonstrating significant advantages in fever reduction, pain relief, phlegm resolution, and cough suppression, with no significant adverse effects (He et al., 2019). Based on these findings, combining YHPG granules with conventional treatment holds promise for a synergistic therapeutic effect in terms of anti-inflammatory, antibacterial, and antiviral effects; immune enhancement; and clinical symptom improvement, thereby offering enhanced therapeutic benefits for CAP.

However, the current body of clinical evidence is insufficient to substantiate effective supplementation of YHPG granules in conjunction with conventional treatment for CAP. Therefore, this clinical trial aimed to verify whether augmenting conventional treatment with YHPG granules would result in superior efficacy and safety compared to conventional treatment alone.

## 2 Materials and methods

### 2.1 Study design

This randomized, double-blind, parallel-controlled clinical trial was conducted at the Hangzhou First People’s Hospital from August 2021 to December 2022. The study protocol was approved by the Ethics Committee of Hangzhou First People’s Hospital (approval no. IRB#2021-20210408-01), and registered with the Chinese Clinical Trial Registry (ChiCTR2100047501). Prior to commencing treatment in this trial, all participants or their legally authorized representatives (in the event that the patients themselves were unable to provide informed consent) provided informed consent and agreed to participate in the study, following the guidelines of the Helsinki Declaration. Furthermore, this study strictly adhered to the CONSORT (Consolidated Standards of Reporting Trials) 2010 guidelines (Schulz et al., 2010).

### 2.2 Inclusion and exclusion criteria for participants

The inclusion criteria were as follows: 1) age between 18 and 85 years; 2) diagnosis of CAP, defined as radiographically confirmed new pulmonary infiltrates and at least one of the following criteria: presence of cough, sputum production, fever, or difficulty in breathing; abnormal auscultation breath sounds or rales; white blood cell count >10 × 10^9/L or <4 × 10^9/L (Chinese Thoracic Society, 2016); and 3) Chinese medicine evidence of evil stagnation of the lung and Wei evidence (ESLWE; Wan et al., 2017). The diagnostic criteria for ESLWE in traditional Chinese medicine (TCM) are as follows: (1) primary symptoms: fever with cold aversion, cough, and poor sweating; (2) secondary symptoms: headache, nasal congestion, runny nose, coughing and sputum, sore throat, and thirst; (3) tongue and pulse: normal tongue texture or red tongue edges, thin white or yellowish moss, and floating pulse. If the subject has “fever with cold aversion + cough” or “fever with cold aversion + poor sweating” with at least two secondary symptoms, and combines with the tongue and pulse, the diagnosis can be made (Wan et al., 2017).

The exclusion criteria were as follows: 1) allergy to botanical drugs in YHPG granules; 2) previous use of other TCM treatments; 3) diagnosis of pulmonary tumors, tuberculosis, or other non-infectious pulmonary diseases; 4) diagnosis of hospital-acquired pneumonia; 5) coexisting severe heart, brain, liver, or kidney diseases; 6) pregnancy, current pregnancy, or breastfeeding; 7) severe mental disorders; 8) participation in concurrent clinical trials during the study period; and 9) unwillingness to adhere to treatment.

### 2.3 Randomization and allocation concealment

A statistician from the collaborating center (Zhejiang Chinese Medical University) performed simple randomization using statistical software (SPSS 26.0) to generate a randomization list, which included observation sequence numbers (1–240) and the corresponding random numbers (1–240), assigning patients to either the YHPG or placebo group based on the random numbers. The independent research coordinator enclosed each patient’s observation sequence number in an opaque envelope, labeling it with the patient’s identifier (enrollment number). The pharmacist performed blinded packaging according to the correspondence between observation sequence numbers and drug numbers in the randomization list. The drug numbers were pre-assigned by Shaanxi Dongke Pharmaceutical Co., Ltd., and efforts were made to ensure that both YHPG granules and the placebo were identical in appearance, taste, and packaging. The pharmacist labeled each drug with a sequence number and recorded the internal correspondence between the sequence numbers and drug numbers. Nurses retrieved the drugs according to the patient’s sequence number and distributed them to the patients. The randomization list was jointly held by the head of the research unit and the sponsor, ensuring that all individuals involved in the trial, including participants, research personnel, healthcare providers involved in drug efficacy and safety assessments, monitors, data managers, and statistical analysts, remained blinded to patient grouping and medication allocation throughout the trial. In case of a severe adverse event during the trial, the investigator must seek approval from the head of the research unit before unblinding becomes permissible.

### 2.4 Interventions

Patients were randomly assigned to two groups: one receiving YHPG granules in addition to conventional treatment (YHPG group), and the other receiving a placebo (mimicking YHPG granule) in addition to conventional treatment (placebo group). Both groups took the medication twice daily, with one sachet each time, continuously for 10 days. Routine treatment involved the use of initial empirical anti-infective drugs, including amoxicillin/clavulanic acid, doxycycline/minocycline, β-lactams, and respiratory quinolones, as recommended by the guidelines (Chinese Thoracic Society, 2016). Throughout the intervention, participants were permitted to adhere to the relevant guidelines for managing comorbid conditions, including hypertension and diabetes.

The trial spanned 8 weeks, comprising a 7-day baseline, a 10-day intervention, and a 56-day follow-up period. Throughout the study, the researchers conducted six follow-up assessments at baseline, 72 h, 5 days, 10 days, 28 days, and 56 days. Laboratory examinations, including complete blood cell count, general urine analysis, liver function, and kidney function, were conducted for each participant at baseline and on the 10th day of follow-up after the trial commenced.

### 2.5 Study medication

YHPG granules consists of *Lonicera japonica* Thunb. [Caprifoliaceae; *Lonicerae japonicae flos*], *Reynoutria japonica* Houtt. [Polygonaceae; *Polygoni cuspidi rhizoma et radix*], *Pueraria montana* var. *lobata (Willd.)* Maesen & S. M. Almeida ex Sanjappa & Predeep [Fabaceae; *Puerariae lobatae radix*], *Ephedra sinica* Stapf. [Ephedraceae; *Ephedrae herba*], *Prunus armeniaca* L. [Rosaceae; *Armeniacae semen amarum*], and *Glycyrrhiza glabra* L. [Fabaceae; *Glycyrrhizae radix et rhizome*] in a ratio of 4:4:4:2:2:1. They were prepared through a series of processes, including extraction, concentration, and drying, resulting in concentrated granules. Each sachet weighed 6 g. Shaanxi Dongke Pharmaceutical Co., Ltd. supplied and manufactured the YHPG granules and placebo. The YHPG granules were certified by the National Medical Products Administration (China; Drug Approval No.: Z20184088), awarded a national invention patent (Patent No. ZL03151188.0), and received a new Chinese medicine certificate (Certificate No. Z20120004). The matched placebo contained 2% YHPG and included edible cornstarch, silicon dioxide, caramel, and sunset yellow. It closely resembles the YHPG granules in terms of odor, color, taste, texture, and packaging.

YHPG was prepared as follows: for every 1,000 g of granules, weigh 1,000 g of *L. japonicae flos*, 1,000 g of *Polygoni cuspidati rhizoma et radix*, 1,000 g of *P. lobatae radix*, 500 g of *E. herba*, 500 g of *A. semen amarum*, and 250 g of *G. radix et rhizome* according to the ratio of the prescription. *Puerariae lobatae radix* and *L. japonicae flos* were extracted by refluxing with 70% ethanol for 12 h and then extracted for two times at 85°C for 1 h each time, and then combined and extracted the solution was centrifuged (15,000 rpm), recovered ethanol under reduced pressure to an extract with relative density of 1.14 (50°C–60°C), and set aside./min), decompression recovery of ethanol to relative density of 1.14 (50°C–60°C) of the extract, standby. *Polygoni cuspidati rhizoma et radix* with 80% ethanol, macerated for 12 h at 85°C reflux extraction two times, each time 1 h, combined extracts, centrifugation (15,000 rpm), decompression recovery of ethanol to relative density of 1.13 (45°C–50°C) of the extract, standby. After soaking *E. herba* and *G. radix et rhizome* in water for 2 h, *A. semen amarum* was added, and the samples were decocted them 3 times for 1 h each time. The decoctions were combined and centrifuged (15,000 rpm). The extracts were concentrated to a relative density of 1.15 (50°C–60°C); the above extracts were combined and dried by spray, and the appropriate amount of dextrin was added to obtain 1,000 g particles.

The YHPG granules samples were analyzed using high performance liquid chromatography (HPLC), and six metabolites were identified by comparison with the standards (Supplementary Material).

### 2.6 Outcome measures

All efficacy endpoints were exploratory. The primary outcome measure was the pneumonia cure rate. Syndrome scores on days 0, 5, and 10 were calculated based on the Chinese syndrome scale, which comprises eight items for assessing the major and minor symptoms of pneumonia. Each major symptom was scored from 0 to 6, and each minor symptom was scored from 0 to 3. Higher scores indicate more severe symptoms (Wan et al., 2017). Efficacy was evaluated using the Nemeroff method (Editor-in-Chief Zheng Xiaoyu, 2002). Pneumonia cure and overall pneumonia efficacy rate were evaluated on the 5th and 10th days.

Secondary outcomes included 1) pneumonia chest absorption rate, defined as the absorption rate of chest CT relative to baseline after treatment, assessed by an independent and experienced radiologist; 2) criticality score (SMART-COP score; Charles et al., 2008); 3) Acute Physiology and Chronic Health Evaluation II (APACHE II) score (Knaus et al., 1985); 4) levels of C-reactive protein (CRP), lactate (LC), and procalcitonin (PCT); 5) time to recovery of symptoms (fever, cough, lung rale) and length of hospital stay; and 6) TCM syndrome scores (including fever, cough, sweating, headache, nasal congestion, runny nose, sputum, sore throat, thirst).

Safety outcomes included monitoring of vital signs, complete blood examination, urine examination, liver and kidney function tests, and adverse events.

### 2.7 Sample size

Based on our team’s prior clinical research on pneumonia treatment (Wan and Jin, 2017; Wan et al., 2017), the cure rate in the treatment group ranged from 85% to 89%, whereas that in the placebo group ranged from 69% to 74%. Assuming an error rate parameter of α = 0.025 for the YHPG group and β = 0.2 for the placebo group, with u_1–0.025_ from the boundary value table as 1.96 and u_1–0.2_ as 0.84, the ratio of the experimental group to the control group was 1:1, using a superiority test. Considering a 10% dropout rate, 240 participants were required for the trial, with 120 participants allocated to each group.

### 2.8 Statistical analysis

Statistical analysis was performed using the SPSS software (version 26.0). Continuous variables following normal distribution are presented as the mean ± standard deviation, whereas non-normally distributed variables are expressed as median (interquartile range) [M (P25, P75)]. For continuous variables that followed a normal distribution, group *t*-tests were used for intergroup comparisons, and paired *t*-tests were used for intragroup comparisons before and after treatment. For severely skewed continuous variables, intergroup comparisons were performed using the Wilcoxon rank-sum test, and intragroup comparisons before and after treatment were conducted using the Wilcoxon signed-rank test. Categorical variables are presented as frequencies (percentages), and intergroup comparisons were conducted using the chi-square or Fisher’s exact test. Given that all analyses are exploratory, nominal *p* values are reported. The statistical significance level was set at *p* < 0.05, and all tests were two-tailed.

## 3 Results

### 3.1 Participants and baseline characteristics

**FIGURE 1:**
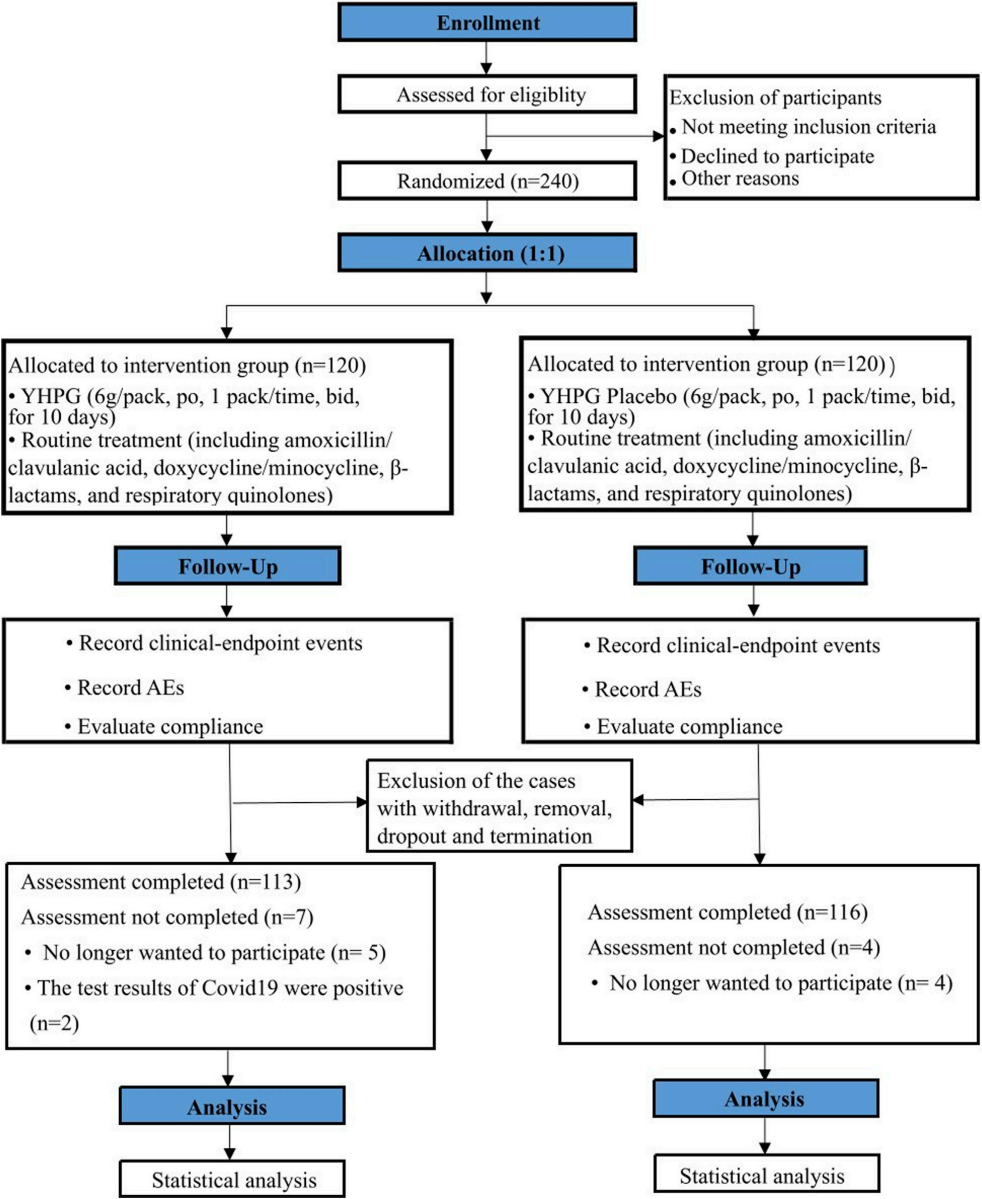
Trial flowchart. Abbreviation: YHPG, Yinhua Pinggan; AE, Adverse event.

Hospitalized patient recruitment occurred from 16 August 2021, to 5 December 2022. A total of 240 eligible participants were randomly allocated to the YHPG or placebo group (n = 120 in each group). After randomization, 11 patients were excluded: 9 patients (five in the YHPG group and four in the placebo group) were lost to follow-up due to discontinuation of medication or testing, and two patients (all in the YHPG group) were excluded from the final analysis because of positive COVID-19 test results. A total of 229 participants completed the clinical trial: 113 in the YHPG group and 116 in the placebo group. A flowchart of the study is presented in Figure 1.

A total of 95 bacterial infections were detected in the 229 cases: 43 in the YHPG group and 52 in the control group (45.3% vs. 54.7%, *p* > 0.05). A total of 48 fungal infections were detected, of which 72.9% were caused by *Candida albicans*. In addition, a small number of patients were infected with pathogenic microorganisms such as *mycoplasma* and viruses. Before treatment, the two groups exhibited comparable baseline demographic characteristics, vital signs, disease severity, SMART-COP scores, APACHE II scores, and TCM syndrome scores (*p* > 0.05; Table 1).

**TABLE 1:** Baseline demographic and clinical characteristics.

Characteristic	YHPG (n = 113)	Placebo (n = 116)	*P* value
Demographics			
Age, mean (SD), y	60.93 ± 13.23	59.51 ± 14.69	0.609
Age ≥ 51, No. (%)	92 (81.42)	87 (75.00)	0.240
Males, No. (%)	49 (43.36)	57 (49.14)	0.381
Vital signs			
Temperature, mean (SD), °C	38.9 ± 0.73	38.6 ± 0.76	0.257
Systolic blood pressure, mean (SD), mmHg	128.06 ± 18.23	129.62 ± 15.22	0.283
Diastolic blood pressure, mean (SD), mmHg	74.58 ± 12.74	75.47 ± 11.31	0.615
Severity, No. (%)			
Non severe	85 (75.22)	90 (77.59)	0.785
Severe	28 (24.78)	26 (22.41)	0.157
SMART-COP score, No. (%)			
Low - risk	75 (66.4)	75 (64.7)	0.785
Moderate - risk	35 (31.0)	37 (31.9)	0.880
High - risk	3 (2.7)	4 (3.4)	0.727
APACHE II score			
Acute physiology score	1.29 ± 1.67	1.76 ± 1.84	0.643
Age score	3.67 ± 1.78	3.35 ± 2.03	0.395
Chronic health condition score	0.61 ± 0.55	0.52 ± 0.32	0.846
Aggregate score	5.57 ± 2.53	5.66 ± 2.18	0.615
TCM syndrome scores	14.32 ± 2.65	14.04 ± 2.41	0.370

Abbreviations: SD, standard deviation; APACHE II, acute physiology and chronic health evaluation II. Continuous data were analyzed using two independent *t*-tests. Categorical variables were examined using the chi-square or Fisher’s exact test.

### 3.2 Cure rate of pneumonia

**FIGURE 2:**
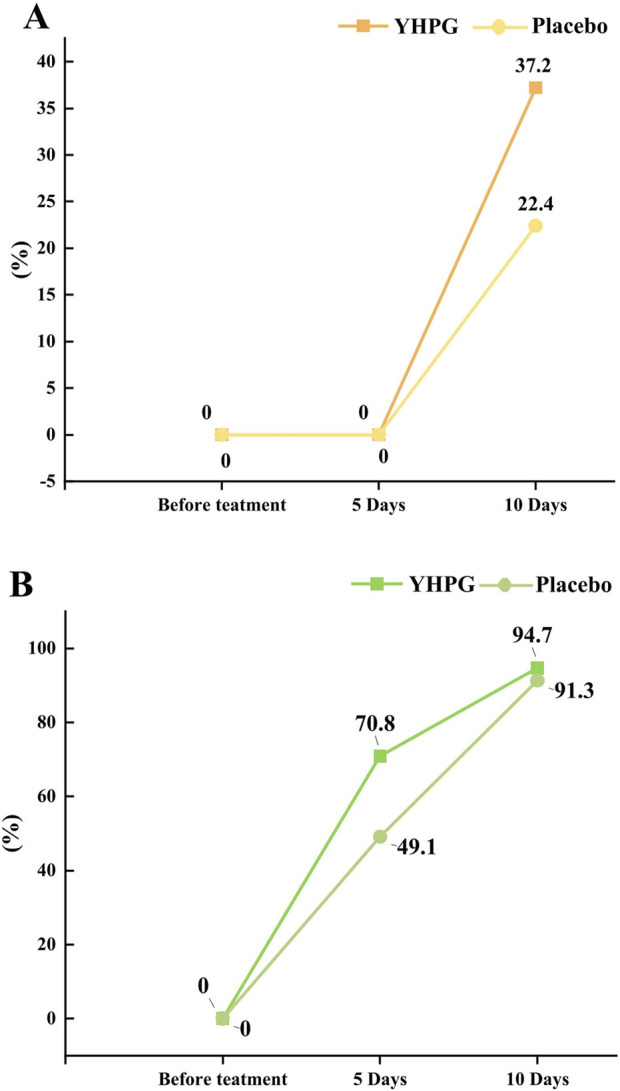
The cure rate of pneumonia between the YHPG and Placebo groups. **(A)** The cure rates; **(B)** the total effective rates; Abbreviation: YHPG, Yinhua Pinggan.

Figures 2A, B show the cure rate and overall effective rate of pneumonia at 0, 5, and 10 days after treatment. After 10 days of treatment, the pneumonia cure rate in the YHPG group surpassed that in the placebo group (37.2% vs. 22.4%, mean difference (MD): 14.75%, 95% CI: 3.05–26.46, *p* = 0.015; Figure 2A). Additionally, patients receiving YHPG granules demonstrated a significantly higher overall pneumonia effective rate than the placebo group after 5 days of treatment (70.8% vs. 49.1%, MD: 21.66%, 95% CI: 9.29–34.03, *p* = 0.001). However, this improvement was not significant on the 10th day (Figure 2B).

### 3.3 Chest CT absorption rate

**FIGURE 3:**
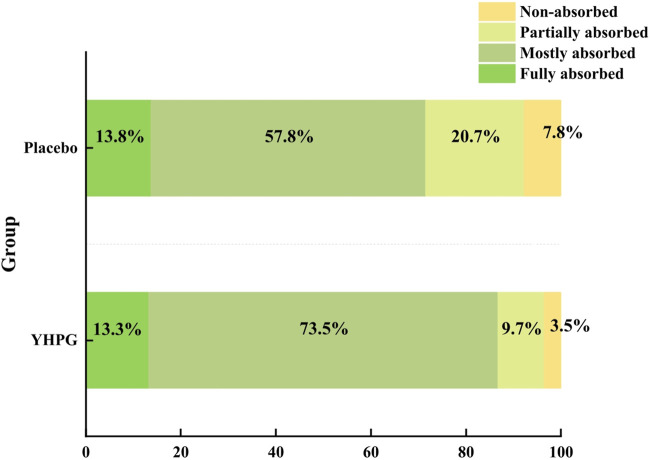
Chest CT absorption rate between the two groups for 10 days of treatment. Abbreviation: YHPG, Yinhua Pinggan.

The degree of absorption on chest CT after drug administration was classified into four grades: “fully absorbed,” “mostly absorbed,” “partially absorbed,” and “non-absorbed.” The percentages of fully and mostly absorbed in the YHPG and control groups were 13.3% vs. 13.8% and 73.5% vs. 57.8%, respectively. Fewer patients had non-absorbed (four in the YHPG group and nine in the placebo group) and partially absorbed (11 in the YHPG group and 24 in the placebo group) CT scans. Compared with the placebo group, the YHPG group had better Chest CT absorption (group comparison or proportion: χ^2^ = 8.453, MD: 15.17%, 95% CI: 4.84–25.50, *p* = 0.049; Figure 3).

### 3.4 SMART-COP score

According to the SMART-COP score, cases were classified into four risk levels: low risk (0–2 points), moderate risk (3–4 points), high risk (5–6 points), and extremely high risk (7–8 points). On the 5th day of treatment, the proportion of low-risk cases in the YHPG group was 90.3% (102/113), which was significantly higher than that in the placebo group (75.0% [87/116]; intergroup comparison or ratio: χ^2^ = 9.253, MD: 5.68%, 95% CI: 15.27–24.86, *p* = 0.002). In the YHPG group, the proportion of moderate-risk cases was 9.7% (11/113), which was significantly lower than that in the placebo group (23.3% [27/116]). However, these improvements were not significant on the 10th day. On the 5th day of treatment, there was no significant difference in the proportion of high-risk cases between the YHPG and the placebo groups (0.0%[0/113] vs. 1.7%[2/116], *p* = 0.489). Additionally, on days 10th, no high-risk cases were observed in either of the groups.

### 3.5 APACHE II score

After 10 days of treatment, there was no significant difference in the total APACHE II score between the YHPG and the placebo groups (4.81 ± 2.24 vs. 4.61 ± 2.07, MD: 1.04%, 95% CI: 0.93–1.17, *p* = 0.478). Similar results were observed in the comparison of acute physiology, age, and chronic health condition scores between the two groups (*p* = 0.229, *p* = 0.316, and *p* = 0.157, respectively; Table 2).

**TABLE 2:** APACHE II score risk assessment between the two groups.

	Category	YHPG	Placebo	Mean difference (95% CI)	*P* value
Before treatment	Acute physiology score	1.29 ± 1.67	1.76 ± 1.84	0.73 (0.54–1.09)	0.643
	Age score	3.67 ± 1.78	3.35 ± 2.03	1.07 (0.90–1.28)	0.316
	Chronic health Status score	0.61 ± 0.55	0.52 ± 0.32	1.17 (0.96–1.43)	0.846
	Aggregate score	5.57 ± 2.53	5.66 ± 2.18	0.98 (0.88–1.21)	0.615
10th days	Acute physiology score	0.95 ± 1.30	1.16 ± 1.77	0.82 (0.56–1.19)	0.229
	Age score	3.67 ± 1.78	3.35 ± 2.03	1.07 (0.90–1.28)	0.316
	Chronic health Status score	0.20 ± 0.32	0.12 ± 0.44	1.67 (0.80–3.46)	0.157
	Aggregate score	4.81 ± 2.24	4.61 ± 2.07	1.04 (0.93–1.17)	0.478

Abbreviation: YHPG, Yinhua Pinggan. Continuous data were analyzed using two independent *t*-tests.

### 3.6 CRP, LC, and PCT

**FIGURE 4:**
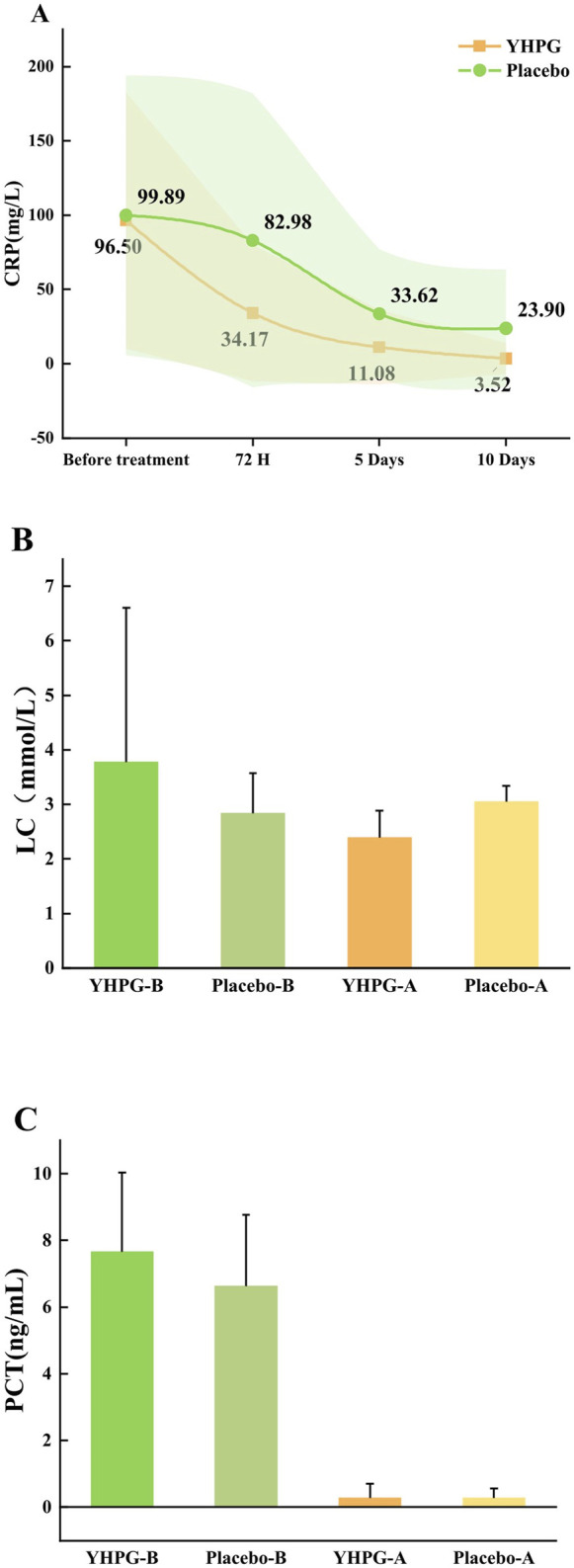
Changes in baseline inflammatory index between two groups. **(A)** CRP; **(B)** LC; **(C)** PCT; YHPG-B, inflammatory index of the YHPG group before the treatment; Placebo-B, inflammatory index of the Placebo group before the treatment; YHPG-A, inflammatory index of the YHPG group after the treatment; Placebo-A, inflammatory index of the Placebo group after the treatment. Abbreviation: YHPG, Yinhua Pinggan.

Figure 4A shows the CRP levels at baseline, 72 h, 5 days, and 10 days. After 72 h and 5 days of treatment, the YHPG group had significantly lower CRP levels than the placebo group (34.17 ± 46.05 vs. 82.98 ± 98.98, MD: 0.41%, 95% CI: 0.30–0.57, *p* = 0.005; 11.08 ± 25.09 vs. 33.62 ± 43.53, MD: 0.33%, 95% CI: 0.20–0.53, *p* = 0.001). However, no significant difference was observed between the two groups after 10 days of treatment (Figure 4A). Additionally, Figures 4B, C show the LC and PCT levels at baseline and after 10 days. After 10 days of treatment, LC levels in the YHPG group were significantly lower than those in the placebo group (2.39 ± 0.49 vs. 3.05 ± 1.01, MD: 0.78%, 95% CI: 0.73–0.84, *p* = 0.005; Figure 4B). However, after 10 days of treatment, no significant difference in PCT levels was observed between the two groups (0.27 ± 0.43 vs. 0.27 ± 0.29, MD: 1.00%, 95% CI: 0.70–1.42, *p* = 0.646; Figure 4C).

### 3.7 Time to recovery of symptoms and length of hospital stay

**FIGURE 5:**
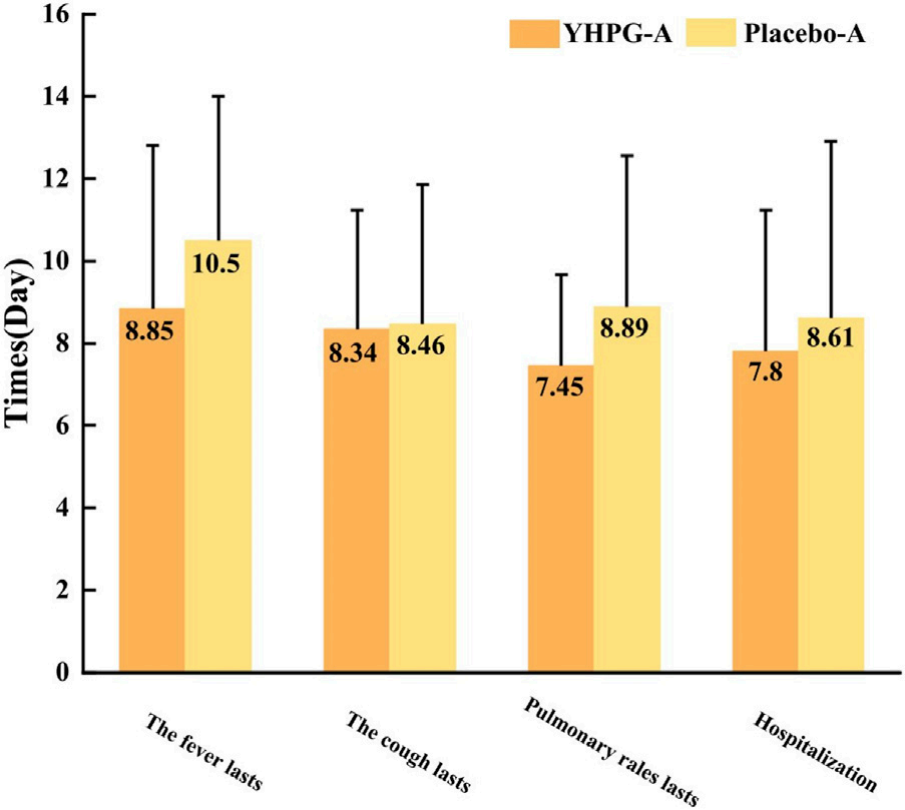
Time to recovery of symptoms and length of hospital stay. YHPG-A, duration of existence of the YHPG group after treatment; placebo-A, duration of existence of the Placebo group after treatment. Abbreviation: YHPG, Yinhua Pinggan.

Figure 5 shows the time to recovery of symptoms (fever, cough, and lung rale) and length of hospital stay. Compared to the placebo group, the YHPG group exhibited a significantly shorter fever recovery time (8.85 ± 3.96 days vs. 10.50 ± 3.51 days, MD: 0.84%, 95% CI: 0.76–0.93, *p* = 0.008) and lung rale recovery time (7.45 ± 2.21 days vs. 8.89 ± 3.68 days, MD: 0.84%, 95% CI: 0.76–0.92, *p* = 0.031). However, no significant differences were observed between the two groups in terms of cough recovery time (8.34 ± 2.91 days vs. 8.46 ± 3.39 days, MD: 0.99%, 95% CI: 0.89–1.09, *p* = 0.623). Furthermore, the length of hospital stay in the YHPG group was significantly shorter than in the placebo group (7.80 ± 3.45 days vs. 8.61 ± 4.31 days, MD: 0.91%, 95% CI: 0.80–1.02, *p* = 0.012; Figure 5).

### 3.8 TCM syndrome scores

On the 5th and 10th days, the TCM syndrome scores of the YHPG group were significantly lower than those of the placebo group (9.67 ± 4.68 vs. 11.89 ± 3.12, MD: 0.81%, 95% CI: 0.74–0.90, p = 0.011; 5.11 ± 3.30 vs. 6.61 ± 2.79, MD: 0.77%, 95% CI: 0.67–0.89, *p* = 0.009; Table 3).

**TABLE 3:** Comparison of total scores of TCM syndromes between the two groups before and after treatment.

Groups	Before treatment	5th days	10th days	*P* value
YHPG	15.61 ± 3.15	9.67 ± 4.68	5.11 ± 3.30	0.000***
Placebo	15.72 ± 3.61	11.89 ± 3.12	6.61 ± 2.79	0.000***
Mean difference (95% CI)	0.99 (0.94–1.05)	0.81 (0.74–0.90)	0.77 (0.67–0.89)	—
*P*	0.675	0.011*	0.009**	—

Abbreviation: YHPG, Yinhua Pinggan. Group *t*-tests were used for intergroup comparisons, and paired *t*-tests were used for intragroup comparisons before and after treatment. *P < 0.05, **P < 0.01 vs. the control group.

### 3.9 Adverse events

The overall incidence of adverse events was not significantly different between groups (*p* = 0.124). The most common adverse events in both groups were elevated neutrophil and aspartate levels. Compared with the control group, we observed a reduction in some adverse events with the use of YHPG, such as hypokalemia, anemia, increased neutrophils, elevated aspartate aminotransferase, and increased D-dimer levels. No severe adverse events were reported during the trial (Table 4).

**TABLE 4:** Summary of adverse events (%).

	Total (n = 229)	YHPG (n = 113)	Placebo (n = 116)	Mean difference (95% CI)	*P* Value
Any	193 (84.3)	91 (80.5)	102 (87.9)	7.40 (−2.00–16.80)	0.124
Hypoalbuminemia	56 (24.5)	22 (19.5)	34 (29.3)	9.84 (−1.20–20.88)	0.083
Hypokalemia	34 (14.8)	8 (7.1)	26 (22.4)	15.33 (24.28–6.39)	0.001**
Increased blood glucose	109 (47.6)	47 (41.6)	62 (53.4)	19.18 (7.34–31.02)	0.073
Anemia	83 (36.2)	33 (29.2)	50 (43.1)	4.24 (11.89–36.60)	0.029*
Thrombocytopenia	9 (3.9)	2 (1.8)	7 (6.0)	4.26 (−0.70–9.23)	0.097
Increased blood lipids	102 (44.5)	43 (38.1)	59 (50.9)	32.02 (22.07–41.96)	0.051
Increased blood urea nitrogen	17 (7.4)	9 (8.0)	8 (6.9)	1.07 (−5.73–7.86)	0.758
Increased neutrophil	128 (55.9)	52 (46.0)	76 (65.5)	19.50 (6.88–32.12)	0.003**
Aspartate aminotransferase increased	111 (48.5)	43 (38.1)	68 (58.6)	20.57 (7.90–33.24)	0.002**
Abnormal serum sodium	26 (11.4)	13 (11.5)	13 (11.2)	0.30 (−7.92–8.52)	0.943
Increased serum potassium	0 (0.0)	0 (0.0)	0 (0.0)	—	—
Increased D-dimer	79 (34.5)	31 (27.4)	48 (41.4)	13.95 (1.78–26.11)	0.026*

Data are presented as n (%) and include all events reported after enrollment. Some patients experienced more than one adverse event. None of the patients discontinued the drug unless they were discharged from hospital or died early. Abbreviation: YHPG, Yinhua Pinggan. Categorical variables were examined using the chi-square or Fisher’s exact test. *P < 0.05, **P < 0.01 vs. the control group.

## 4 Discussion

Research on botanical preparations has demonstrated their significant benefits in the treatment of pneumonia. However, these studies are often limited by factors such as small sample size (Zheng et al., 2022), fewer observed indicators (Li et al., 2015; Zheng et al., 2022), and short follow-up duration (Xu et al., 2018). Given the limited prescription of botanical drugs recommended in the practical guidelines for treating CAP in China, optimizing the clinical management and treatment strategies for CAP holds significant value. This randomized controlled trial represents the first exploration of YHPG as an adjunct treatment for CAP. This RCT affirms the efficacy, safety, and superiority of the botanical preparation of YHPG granules in treating patients with CAP.

### 4.1 TCM botanical drugs and theory of YHPG granules

YHPG is composed of six botanical drugs: *E. herba*, *A. semen amarum*, *L. japonicae flos*, *P. cuspidati rhizoma et radix*, *P. lobatae radix*, and *G. radix et rhizoma*.

*Lonicerae japonicae flos,* which was first recorded in the *Records of Famous Doctors* (edited in the late Han dynasty), is the dried flower bud of the *L. japonica* Thunb. plant. It is sweet in taste and cold in nature, and belongs to the lung, heart, and stomach meridians. It is known for its efficacy in clearing heat, removing toxins, and evacuating wind-heat, and is often used in the treatment of wind-heat colds, fevers of warm diseases, and carbuncles and boils. Because of its aromatic and evacuating properties, *L. japonicae flos* is good at clearing evils in the lung meridian to clear wind and penetrate heat, and it is the main botanical drug in YHPG Granules for the treatment of CAP ESLWE. According to the classic work of Chinese medicine, *Item Differentiation of Warm Febrile Diseases* (edited by Wu, J. T. in 1798), when the disease is located in the lungs, treatment should involve the use of products that are light, clearing, propagating, and dispersing.

*Polygoni cuspidati rhizoma et radix* is the dried root and rhizome of the plant *R. japonica* Houtt. It is slightly cold in nature, bitter in taste, and belongs to the liver, gallbladder, and lung meridians. It can clear the lungs, resolve phlegm, and stop coughing and is often used to treat lung-heat cough.

*Puerariae lobatae radix* is the dried root of the plant *P. montana* var. *lobata (Willd.)* Maesen & S.M.Almeida ex Sanjappa & Predeep. It is cool in nature, sweet and pungent in taste, located in the Spleen, Stomach and Lung meridians, and is effective in relieving fever, quenching thirst, penetrating rashes, and stopping diarrhea. According to the *Materia Medica Zheng* (edited by Zhang, J. B.in 1624), it is good at “relieving surface and sweating” and is a good medicine for treating wind-heat.

*Ephedrae herba* is the dried root and rhizome of the *E. sinica* Stapf. plant. It is pungent, slightly bitter, and warm in nature, with the efficacy of sweating and relieving symptoms, promoting the lungs to calm asthma, and inducing diuresis to reduce edema. It mainly enters the lung meridian and is the treatment of lung qi congestion caused by wheezing and coughing of the important medicine clinically used in the treatment of wind-cold surface evidence, cough, and asthma.

*Armeniacae semen amarum* is the dried mature seed of the *P. armeniaca* L. plant. It is bitter in taste, slightly warm in nature, and belongs to the lung and large intestine meridians, relieves cough and asthma, and has laxative effects.

*Glycyrrhizae radix et rhizome* is the dried root and rhizome of the *G. glabra* L.plant. It is sweet in taste, flat in nature, and belongs to the heart, lungs, spleen, and stomach meridians. According to the *Compendium of Materia Medica* (edited by Li in 1578), it can tonify qi, dispel phlegm, relieve cough, detoxify, relieve pain, and harmonize all medicines.

### 4.2 Modern pharmacological research on the active metabolites of YHPG granules

YHPG particles composed of different botanical drugs may enhance the clinical efficacy of CAP *via* multiple targets and pathways. The modern pharmacological analyses of the active metabolites of YHPG granules are as follows.

*Lonicerae japonicae flos* has anti-inflammatory (Kao et al., 2015; Park et al., 2014; Yoo et al., 2008), anti-angiogenic, anti-nociceptive (Yoo et al., 2008), and antioxidant (Lan et al., 2007) activities. In a study (Kao et al., 2015), *L. japonicae flos* was shown to reduce acute pulmonary inflammation induced by lipopolysaccharides, with its potential mechanism involving the downregulation of pro-inflammatory cytokines (TNFα, IL-1β, and IL-6) and upregulation of anti-inflammatory cytokine IL-10 expression. Our study used HPLC to determine the chemical composition of Honeysuckle and determined that its main metabolite is chlorogenic acid. Chlorogenic acid was shown to inactivate the p38MAPK pathway by increasing the expression of miR-124-3p, thereby alleviating *Klebsiella* pneumoniae-induced lung inflammation (Zhang et al., 2023).

Resveratrol, the main metabolite of *P. cuspidati rhizoma et radix*, exhibits strong antibacterial activity (Vestergaard and Ingmer, 2019) and significantly enhances the susceptibility of multidrug-resistant *K. pneumoniae* to polymyxin B (Liu et al., 2020). Furthermore, resveratrol can reverse oxidative stress induced by *S. pneumoniae* in lung epithelial cells, thereby reducing lung inflammation and damage (Zahlten et al., 2015).

According to HPLC compositional analysis, puerarin was the most concentrated metabolite in the YHPG granules. Puerarin is the primary active metabolite of *P. lobatae radix* and exhibits a wide range of pharmacological effects, including analgesic, antipyretic, anti-inflammatory, antioxidant, and anticancer properties (Zhou et al., 2014). In addition, Puerarin can treat acute viral respiratory diseases by inhibiting the replication of the influenza virus, which is achieved by blocking the nuclear export of the viral ribonucleoprotein (Wu et al., 2011).

*Ephedrae herba* and *A. semen amarum* form a classic botanical combination for treating pneumonia. Key active metabolites of this pair of botanical drugs, including quercetin, kaempferol, and bergenin, may combat pneumonia by inhibiting inflammation, demonstrating antiviral activity, and immune system regulation. These metabolites may synergistically function through the PI3K-Akt, IL-17, and TNF signaling pathways (Gao et al., 2020). Moreover, the combination of *E. herba* and *G. radix et rhizoma* has antiviral, immunomodulatory, and organ-protective effects, possibly linked to the activation of the PI3K/Akt signaling pathway (Li et al., 2021).

### 4.3 Clinical efficacy and safety of YHPG granules in the treatment of CAP ESLWE

In terms of the main therapeutic index of this study, the results showed that the cure rate of pneumonia was significantly higher in the YHPG group than in the control group after 10 days of administration (*p* < 0.05). This indicates that after 10 days of administration, the YHPG group was more effective than the control group. In addition, the total effective rate of the YHPG group was 70.8% after 5 days of administration, which was significantly better than that of the control group (*p* < 0.05). However, with the increase of treatment time, after 10 days of treatment, the total effective rate of the two groups showed an increasing trend, but the difference between the two groups was not statistically significant (*p* > 0.05).

A recent study reported that the cure rate of Ma Xing Shi Gan Tang for the treatment of CAP was significantly higher than that of placebo (22.22% vs. 11.43%; Zheng et al., 2022). Ma Xing Shi Gan Tang and YHPG granules contain *E. herba*, *Armeniacae Semen Amarum*, and *G. radix et rhizome* as their core botanical drugs. In contrast to the study by Ma Xing Shi Gan Tang, our research expanded the analysis to include two additional key outcomes: the criticality score (SMART-COP score) and inflammatory markers. Previous studies have scrutinized existing pneumonia severity assessment tools, such as the pneumonia severity index pneumonia severity index (PSI) and CURB-65, revealing their limited predictive ability in determining the necessity of intensive care unit (ICU) admission (Buising et al., 2006; Capelastegui et al., 2006). In contrast, SMART-COP can accurately identify patients with pneumonia who require enhanced respiratory or vasopressor support, making it an ideal tool for clinicians to determine pneumonia severity (Charles et al., 2008). CRP is the most commonly used biomarker for assessing inflammation and identifying patients with adverse prognoses (Moreno et al., 2010; Ticinesi et al., 2017). Because CRP is a relatively rapid indicator, we added two time points: the CRP level observed at 72 h and on the 5th day. This study aimed to ascertain whether YHPG granules could alleviate inflammation in the short term. If short-term CRP assessment proves effective, the treatment plan may remain unchanged, continuing with previous treatment. Additionally, LC is an important indicator for assessing tissue hypoperfusion and hypoxia, showing reliable predictive performance for the 28-day mortality, hospitalization, and ICU admission rates of patients with pneumonia (Chen and Li, 2015).

In this trial, we observed that the CRP levels of patients in the YHPG group were significantly lower than those in the placebo group after 72 h and 5 days of treatment. Furthermore, after 10 days of treatment, the LC levels of patients in the YHPG group were significantly lower than those in the placebo group. Combined with the chest CT absorption rate results, we observed a significantly higher degree of chest CT absorption in the YHPG group than in the control group (CT absorption rate: 73.5% vs. 57.8%, *P* < 0.05). These results support our hypothesis that supplementing conventional treatments with YHPG granules is effective in promoting the absorption of inflammatory lesions in the lungs and improving CRP and LC levels.The total TCM syndrome scores of the YHPG group were lower than those of the control group at 5 and 10 days after administration (P < 0.05) and showed a decreasing trend with the prolongation of the medication time, which reflected the beneficial effect of YHPG granules on patients with CAP ESLWE. Additionally, YHPG granules were found to be associated with shorter recovery times from fever and lung rales, as well as shorter hospital stays compared to placebo. Therefore, supplementing conventional therapy with YHPG granules may promote faster symptom recovery and earlier discharge from the hospital.

Monitoring of hematological indicators before and after treatment in both groups showed no significant difference in the overall incidence of AEs between the two groups (80.5% in the YHPG group and 87.9% in the control group, P > 0.05). No serious adverse events were observed during drug administration in either group, suggesting that drug safety was high in both groups.

### 4.4 Limitation

Our study had a few limitations. First, this study was exclusively conducted at a medical center in Zhejiang Province, China, potentially limiting the external validity of the results. A multicenter, rigorously controlled randomized trial is required to enhance the generalizability and credibility of the results. Second, in this trial, physicians determined that combination therapy with drugs had anti-infection, expectorant, or antipyretic effects based on the patient’s condition. Consequently, the combination treatment was not uniform. Third, the low detection rate of pathogenic microorganisms in patients with CAP in this study may be due to the lack of standardization in the collection, storage, and transportation of microbiological specimens, as well as the limited range of testing methods used. Metagenomic next-generation sequencing can provide information on the microbial species and flavor present in the specimens. However, because it is not a routine clinical item, it is less commonly used in clinical diagnosis. To determine why YHPG particles are effective in treating pneumonia, future studies will use metagenomic next-generation sequencing to identify a broad range of pathogens and perform subgroup analyses of bacterial, viral, or other infections.

## 5 Conclusion

When used as an adjunct treatment in Western medicine, YHPG granules can significantly increase the pneumonia cure rate and chest CT absorption rate, reduce pneumonia severity and inflammation levels, and improve clinical symptoms in patients. Furthermore, the YHPG granules demonstrated good tolerance and safety. Therefore, YHPG granules are a promising complementary therapy for CAP.

## Data Availability

The original contributions presented in the study are included in the article/Supplementary Material, further inquiries can be directed to the corresponding authors.
