# Supplementary material for: Effectiveness of Yinhua Pinggan granules in community-acquired pneumonia: a randomized, double-blind clinical trial
Source: Front Pharmacol. 2025 Mar 4;16:1446319. doi: 10.3389/fphar.2025.1446319 (PMC11914909; doi:10.3389/fphar.2025.1446319)
Supplement: Supplementary file 1 [file DataSheet1.docx]

**Supporting Information**

Effectiveness of Yinhua Pinggan Granules in Community-Acquired Pneumonia: A Randomized, Double-Blind Clinical Trial

Jiao-Li Wang ^1†^, Hao-Ran Hu ^2†^, Yi-Lei Guo ^2†^, Jin Han ^2*^, Hai-Tong Wan ^2,3*^, Yu-Xiao Tong ^1^, Man Luo ^1^, Xian-Wen Li ^4^

^1^ Department of Respiratory Medicine, Affiliated Hangzhou First People’s Hospital, School of Medicine, Westlake University, Hangzhou, China

^2^College of Basic Medical Science, Zhejiang Chinese Medical University, Hangzhou, China
^3^ Academy of Chinese Medical Sciences, Henan University of Chinese Medicine, Zhengzhou, China
^4^School of Nursing, Hangzhou Medical College, Hangzhou, China

^†^These authors have contributed equally to this work.

* Correspondence:
Jin Han

[3232168@163.com](mailto:3232168@163.com);

Hai-Tong Wan

[whtong@126.com](mailto:whtong@126.com)

**YHPG extraction solution**


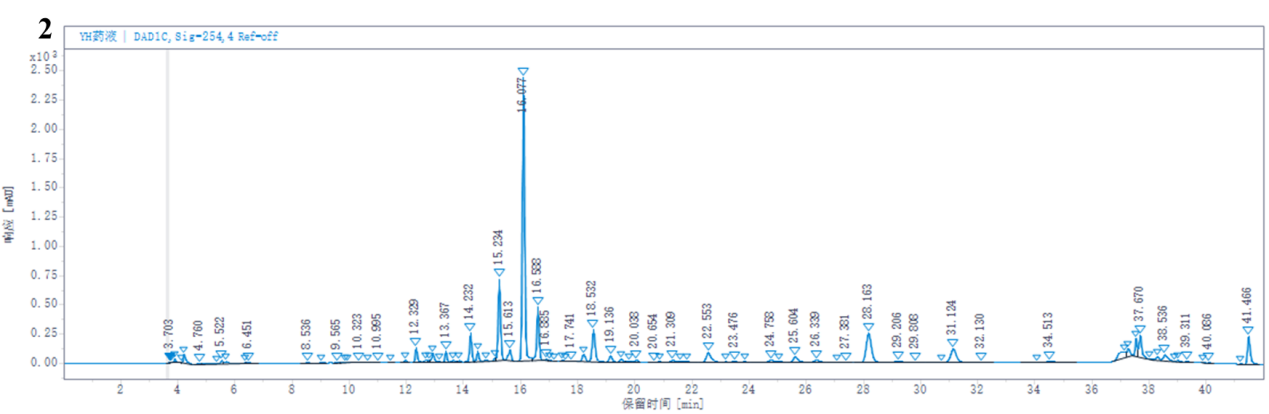

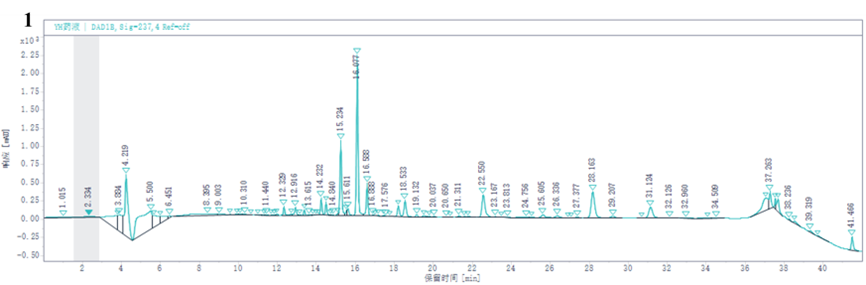


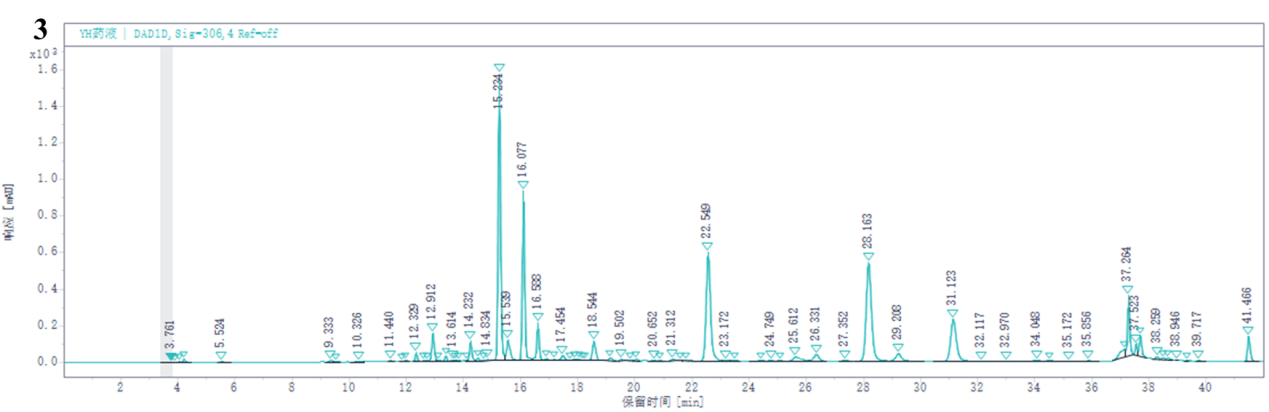


The HPLC chromatogram of YHPG. 1.wavelength = 237 nm; 2. wavelength = 254 nm; 3. wavelength = 306 nm;


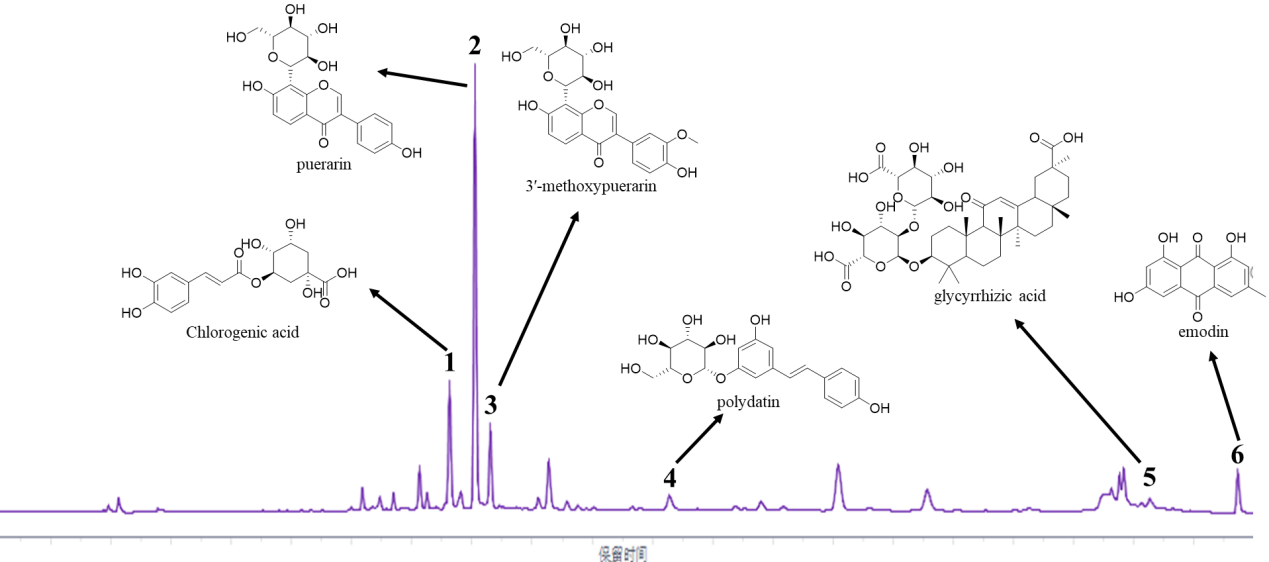


The HPLC chromatogram of YHPG (wavelength = 254 nm). 1. Chlorogenic acid; 2. puerarin; 3. 3′-methoxypuerarin; 4. polydatin; 5. glycyrrhizic acid; 6. emodin.

| Components | Content(mg/g) |
| --- | --- |
| Chlorogenic acid | 34.15±1.25 |
| puerarin | 28.30±1.09 |
| 3′-methoxypuerarin | 9.63±0.12 |
| polydatin | 10.83±0.57 |
| glycyrrhizic acid | 3.33±0.56 |
| emodin | 4.14±0.34 |
